# Utilizing Light-field Imaging Technology in Neurosurgery

**DOI:** 10.7759/cureus.2459

**Published:** 2018-04-10

**Authors:** Brian R Chen, Ian A Buchanan, Spencer Kellis, Daniel Kramer, Ifije Ohiorhenuan, Zack Blumenfeld, Dominic J Grisafe II, Michael F Barbaro, Angad S Gogia, James Y Lu, Beverly B Chen, Brian Lee

**Affiliations:** 1 Department of Neurosurgery, University of Southern California, Los Angeles, USA

**Keywords:** light-field, plenoptic, photography, lytro®

## Abstract

Traditional still cameras can only focus on a single plane for each image while rendering everything outside of that plane out of focus. However, new light-field imaging technology makes it possible to adjust the focus plane after an image has already been captured. This technology allows the viewer to interactively explore an image with objects and anatomy at varying depths and clearly focus on any feature of interest by selecting that location during post-capture viewing. These images with adjustable focus can serve as valuable educational tools for neurosurgical residents. We explore the utility of light-field cameras and review their strengths and limitations compared to other conventional types of imaging. The strength of light-field images is the adjustable focus, as opposed to the fixed-focus of traditional photography and video. A light-field image also is interactive by nature, as it requires the viewer to select the plane of focus and helps with visualizing the three-dimensional anatomy of an image. Limitations include the relatively low resolution of light-field images compared to traditional photography and video. Although light-field imaging is still in its infancy, there are several potential uses for the technology to complement traditional still photography and videography in neurosurgical education.

## Introduction and background

The way we acquire and learn new information is changing rapidly. Learning has become multimodal, with various multimedia technologies allowing the novice to interact with difficult subject material in a way not possible via printed text [1-4]. Today, the learner reasonably expects to peruse high-quality photographs in journals and reference books. The advent of high-quality inexpensive video recording equipment and the proliferation of video sharing technologies has made neurosurgical content easier to consume. Stereoscopic three-dimensional (3D) imaging has experienced a resurgence and enables the viewer to experience enhanced stereoscopic 3D depth perception in still and moving pictures [3, 5-7]. These technologies allow the neurosurgery resident to assimilate large amounts of information in a comprehensive manner not previously possible. A new imaging technology, light-field imaging, also known as plenoptic imaging, allows the user to focus on any part of an image after the image has been captured and may prove to be a valuable tool for neurosurgery education.

## Review

A problem of depth

Neurosurgical anatomy is complex, with deep surgical corridors and vital structures at multiple depths. One fundamental problem facing neuroanatomical imaging is the difficulty in capturing critical depth information using conventional still photography, video, and even stereoscopic 3D imaging, and translating this information to the viewer in an accurate and intuitive manner [8].

Conventional imaging still photography

Conventional still photography has long been utilized to capture images of neurosurgical anatomy and surgical techniques. However, there are significant limitations with conventional still photography. First, the photographer must set the focus at the time of image capture, and focus is fixed to a single distance from the camera. Objects in the photograph that intersect this plane are maximally clear and sharp, and the quality of the lens determines the degree to which the focal plane is flat (i.e., a true two-dimensional surface normal to the vector extending from the camera to the focal point) or curved (i.e., a concave surface where the periphery bends in toward the camera).

Although the focal plane defines the point of maximum sharpness, the aperture, or opening size, of the lens diaphragm determines the depth at which objects in the photograph appear acceptably sharp. For instance, a large aperture creates a very shallow “depth of field,” with objects immediately anterior and posterior to the focus distance appearing out of focus. Photographers typically use a large aperture to create a sense of depth, as the out-of-focus areas provide the viewer contextual clues for discriminating between the foreground and background within a scene. However, as the focus cannot be adjusted after the capture of conventional photographs, the viewer cannot clearly discern any details in these out of focus areas. Focusing an image with a shallow depth of field is also prone to user or equipment error, as slight changes in focus can render the specific feature of interest, or even the entire image, out of focus. By changing the lens aperture to a smaller setting, the depth of field of an image is enlarged so objects near, but not necessarily directly in, the focus plane can appear in focus as well. The shortcoming with this technique is that when everything in an image is in focus, it is more difficult to estimate depth accurately since the photo appears “flat” [9]. A smaller lens aperture decreases light entering the camera for a given exposure time. To correct for the reduction in light, either the shutter speed must be decreased or the sensitivity of the photographical medium (e.g., the ISO rating of the film or digital sensor) must be increased. Both options are undesirable - slow shutter speeds lead to motion blur and higher sensitivity leads to increased noise - and result in obscured photographic detail.

Video

Videos are sequential photographs displayed at multiple frames per second. An advantage of video over still photography is that the focus can be adjusted to highlight various structures as the video is captured. However, as in still photography, the focus point must be set during the time of capture and the viewer cannot change this focus once the video is recorded. Videos are also less practical than still photographs, as they take more time to capture and view while requiring additional storage space and computer equipment.

3D Imaging

Stereoscopic 3D photos and video are gaining popularity in neurosurgical education due to the additional depth information obtained by having two cameras stereoscopically capture a scene from two slightly offset perspectives, thereby simulating human binocular vision. Although there is stereoscopic depth information in 3D photos, these images suffer from the same problem as still photography and video: the focus plane in the picture or video is set during time of capture and cannot be changed by the viewer afterward [10-12].

Light-field imaging

**Figure 1:**
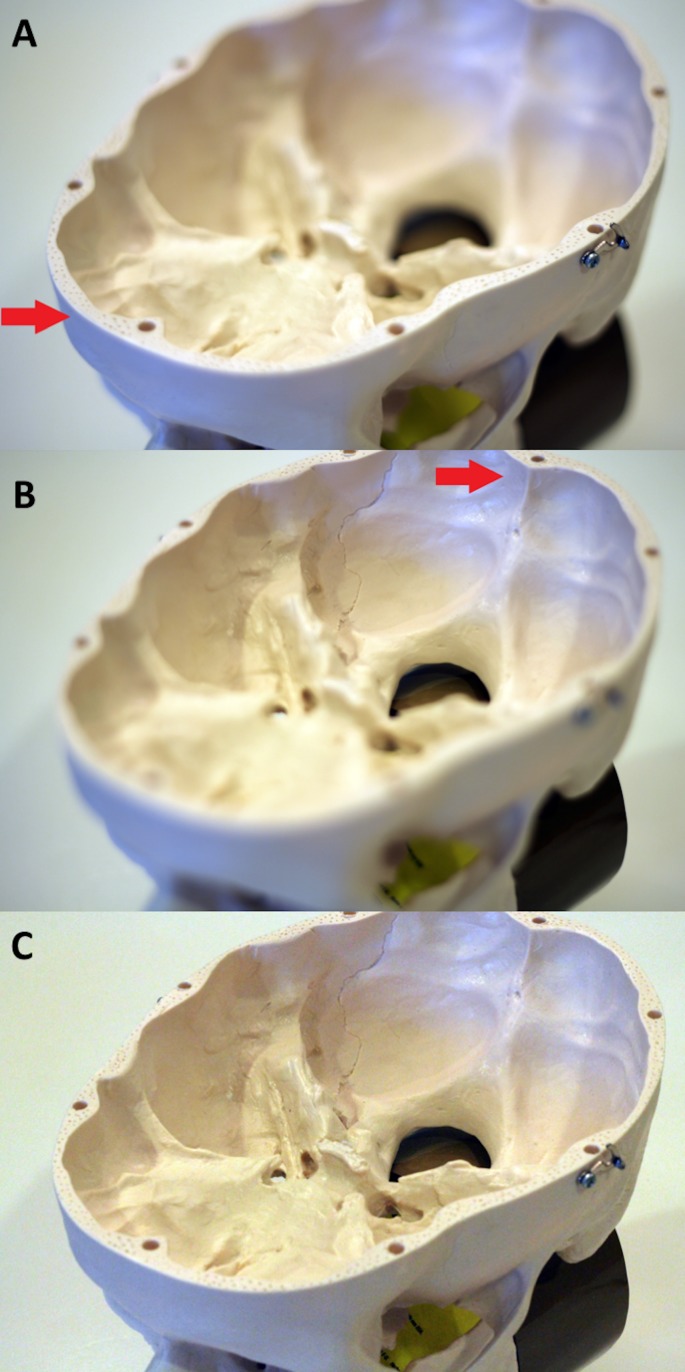
Skull base model with a DSLR camera

**Figure 2:**
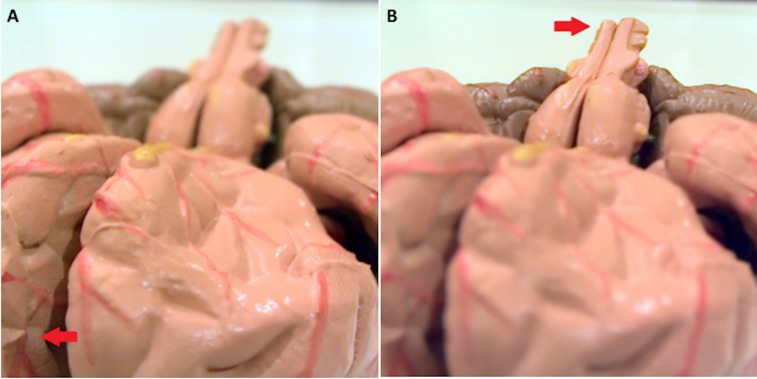
Brain model with a light-field camera

**Figure 3:**
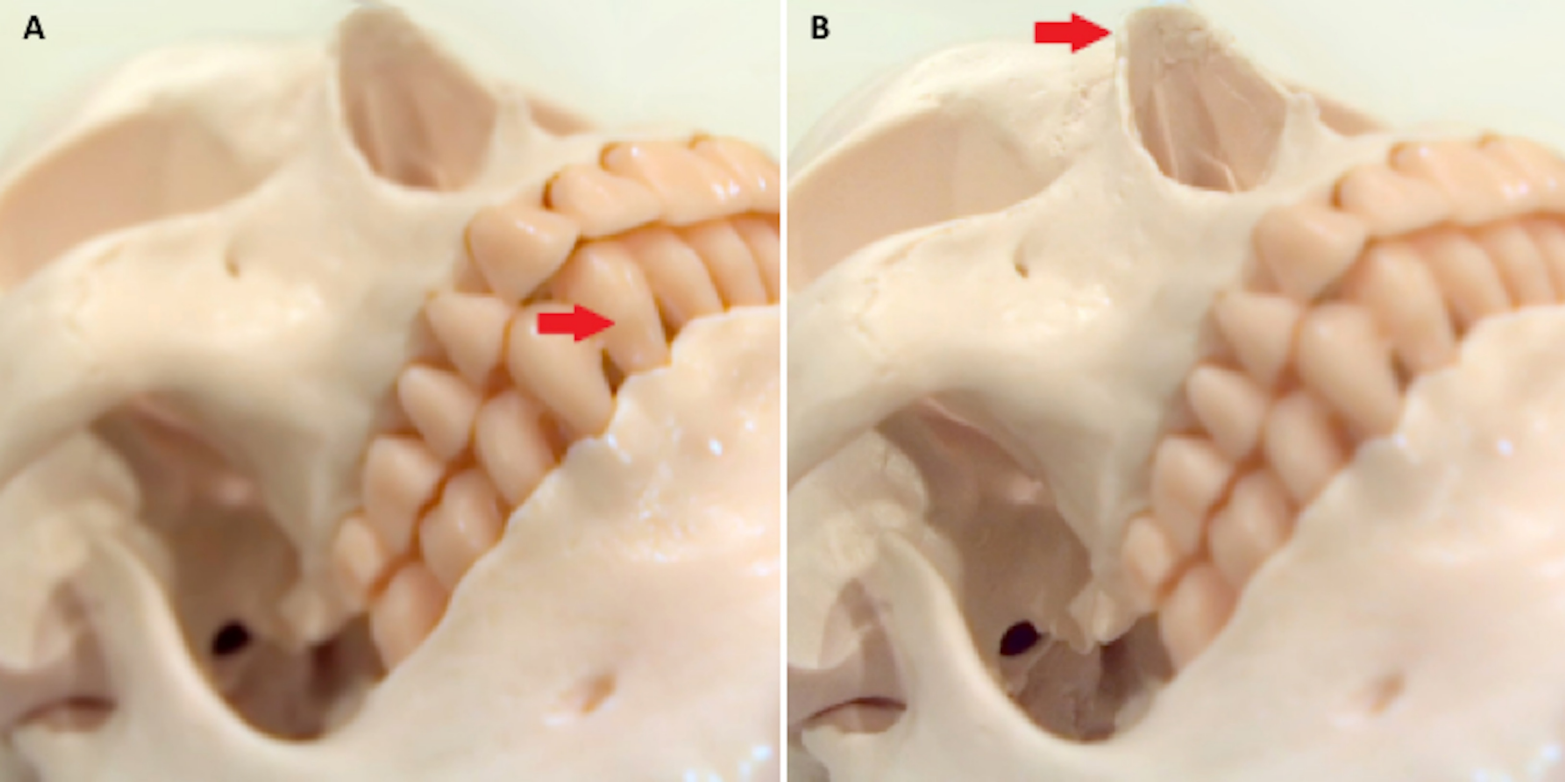
Skull model with a light-field camera

**Figure 4:**
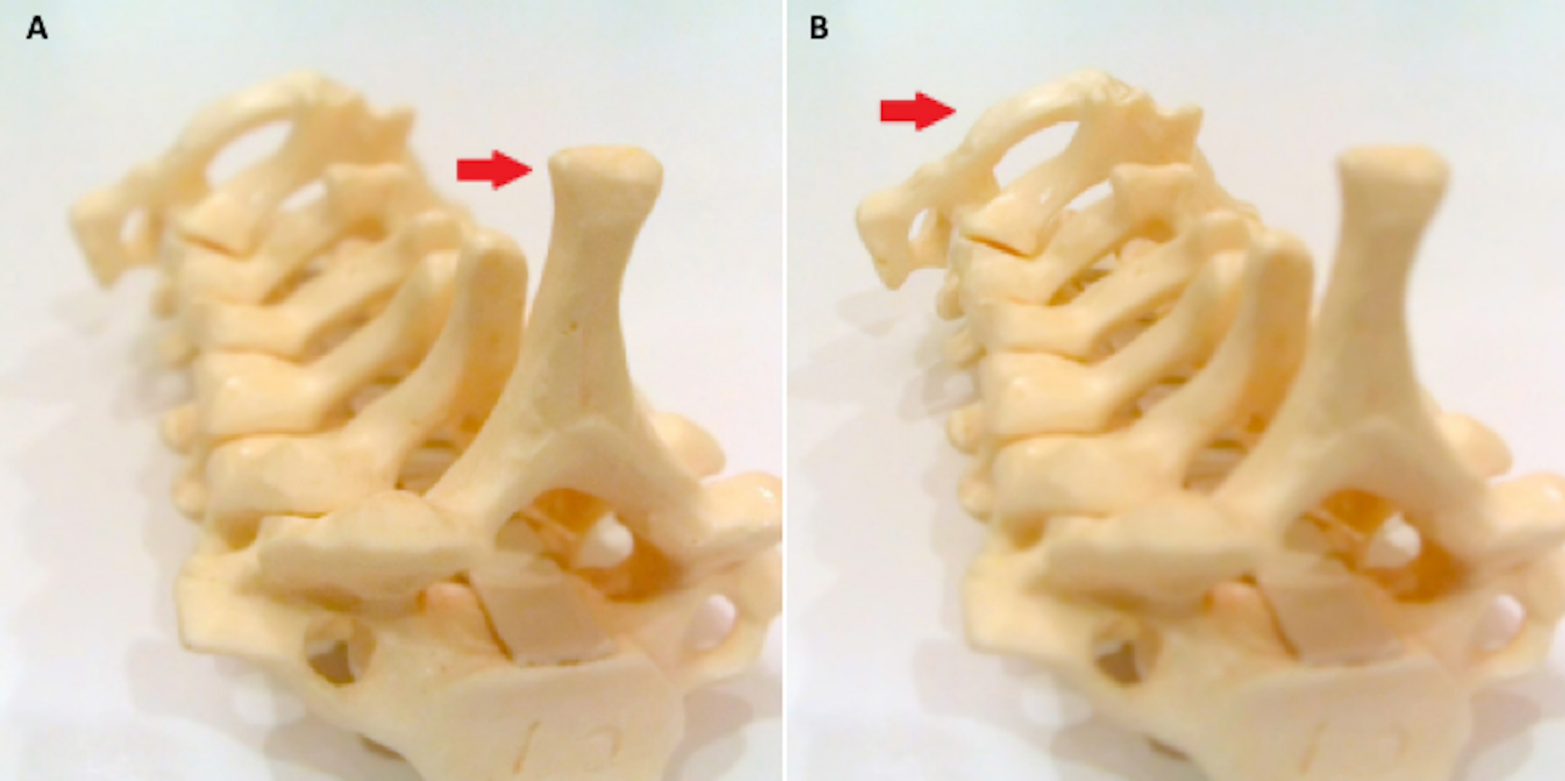
Cervical spine model with a light-field camera

One of the newest developments in optical imaging allows the viewer to change the focus of a photograph after it has already been captured. This is a revolutionary change from previous imaging modalities in which the focus point, and therefore focal plane, is set before the image is captured. This is accomplished by utilizing a light-field camera, also known as a plenoptic camera. This type of camera differs from other cameras in the way light is captured [13]. In most cameras, the light path enters the lens and the resultant image is captured onto film or a digital sensor [14]. A light-field camera captures much more information about the light, including direction, angle, intensity, and color [13, 15]. This additional information is obtained by placing a microlens array in front of the camera sensor. Sophisticated processing of these additional features of the incoming light field allows one to create an image that can have its plane of focus adjusted after the image has been recorded [15]. See Figures 1-4 below. Specific softwares allows the viewer to vary the focal plane of light-field images by clicking on the region of desired focus. Light-field cameras are intuitive to operate, with automatic exposure settings and form factors comparable to existing camera types (e.g., “point-and-shoot” cameras and DSLRs). The user need not adjust focus prior to image capture, as the focus is adjusted in post-processing. With automatic exposure settings and post-capture focusing, the user can rapidly capture images in a variety of circumstances with minimal photographic training and experience. See Table 1 below for a history of light-field imaging.

*Table 1 – Abbreviated history of events leading to* *commercial* *light-field imaging*

**Table 1:** Abbreviated history of light-field-imaging

Year	Milestone
1908	Concept for first light-field camera using integral photography was proposed by Gabriel Lippmann.
1992	Adelson and Wang proposed design of plenoptic camera using array of microlenses [13]
1999	Okana et al. develop three-dimensional video system with a gradient-index lens array based on integral photography [14]
2004	Stanford University Computer Graphics Laboratory used 16 megapixel camera with 90,000 microlens array to demonstrate images that could be refocused after capture [15]
2010	Raytrix begins selling plenoptic cameras for industrial and scientific purposes.
2011	Lytro® sells first commercially available light-field camera to consumers.

Light-field imaging in neurosurgery

There are several potential uses for light-field imaging in neurosurgery. This technology is well-suited for capturing images where the objects of interest might not be located along the same plane, such as when operating in a deep corridor for pineal tumors or down a dilation tube in minimally invasive spine surgery. The freedom to change the plane of focus of the captured image allows the viewer to clearly see the anatomy, regardless of the plane in which the object is located. It also allows the viewer to be an active participant in interacting with the subject, instead of passively viewing the image (Figure 1-4).

Figure 1 - Skull base model with a DSLR camera

Photographs of a skull base model using a conventional digital single-lens reflex (DSLR) camera with a 50mm f/1.8 lens. Note that each image (A, B, C) is an individual exposure with different focus points. In (A) (near focus) and (B) (far focus) a large aperture setting (f/1.8) results in a shallow depth of field. Everything outside this plane is out-of-focus. In (C) (middle focus), set at the dorsum sellae, using a small aperture setting (f/11) increases the depth of field. The entire image, including regions anterior and posterior to the dorsum sellae, appears in focus, which results in the image looking “flat” and lacking depth. Red arrows indicate focus plane.

Figure 2 – Brain Model with a Light-field Camera

Photograph taken with a light-field camera (Lytro® Light Field Camera, Mountain View, CA). Note that each image of the brain model (A, near focus; and B, far focus) is taken from a single exposure. Using software supplied by the light-field camera manufacturer, the photo’s focus point was adjusted after the photo was taken and exported as individual images. Red arrows indicate focus plane. Considering the low resolution of the camera, the model was placed close to the camera lens to capture maximum detail.

Figure 3 – Skull Model with a Light-field Camera

Photograph taken with a light-field camera (Lytro® Light Field Camera). A) Near focus. B) Far focus. Red arrows indicate focus plane.

Figure 4 - Cervical Spine Model with a Light-field Camera

Photograph taken with a light-field camera (Lytro® Light Field Camera). A) Near focus. B) Far focus. Red arrows indicate focus plane.

The same image, if taken by a conventional camera, would only have a single plane in focus. If video were used, the focus could be changed during video recording; however, the viewer must scroll through the video to look for the point when the object of interest was in focus. If the desired object was never focused upon while recording the video, the viewer could not see it. With a light-field image of the same object, the user can direct the focus point to any location they desire.

Limitations

In its current form, light-field cameras have significant limitations compared to conventional imaging techniques. The most outstanding issue is the relatively low resolution of the image. Compared to currently available digital cameras used for conventional still photography, light-field cameras produce lower-resolution images. For instance, the Lytro® (Mountain View, CA, US) Illum light-field camera produces a relatively small 2450 x 1634 pixel image, which is approximately 4.0 megapixels. For reference, at 4.0 megapixels, a photo could be printed at 8 x 5 inches at 300 pixels per inch. Low resolutions prevent any meaningful magnification of the image due to significant loss of detail. This issue is partially addressed by the optical zoom capabilities of the camera lens. However, this limitation will likely be addressed as technology improves and subsequent generations of light-field cameras are developed.

Processing of light-field images is software dependent, and the current Lytro® software application is slow at processing each light-field image compared to traditional images. However, the software is evolving to bring new features to manipulate the light-field in interesting ways. Future software updates may allow for faster processing, higher resolutions, and even stereoscopic 3D capture of images from a light-field data file. See Table 2 for a comparison of conventional DSLR camera with a light-field camera.

Table 2 – Comparison of conventional DSLR camera versus available light-field camera (Lytro® Illum)

**Table 2:** Comparison of conventional DSLR camera versus available light-field camera (Lytro® Illum)

	Conventional DSLR camera	Light-field camera (Lytro® Illum)
Focus	Set at time of image capture	Set after image capture
Interactive photo	Still image on computer	Can change focus point to any location
Optical zoom	1x to 20x depending on lens	8x
Interchangeable lens	Yes	No
Resolution	Up to 50 megapixels	4.0 megapixels
Manual control	Full manual to full automatic	Full manual to full automatic
Can capture 3D information	Yes, with special adapters	Image information contains 3D information in light-field data
Video	Yes, for most cameras	No
Flash	External option	External option

## Conclusions

Light-field imaging holds much promise as a tool neurosurgeons can use to capture meaningful information from the operating room and teach the next generation of neurosurgeons in a way that is interactive and innovative. It is a device beginning to garner interest where accurate imaging of multi-plane anatomy is important, such as the scalp and oral cavity. Capturing complex neuroanatomy using conventional still photography, videos, and stereoscopic 3D imaging is difficult due to the fixed-focus nature of these methods. Light-field imaging has the potential to be a worthwhile companion to conventional still photography.
